# Characterization of apoptosis and autophagy through Bcl-2 and Beclin-1 immunoexpression in gestational trophoblastic disease

**Published:** 2015-07

**Authors:** Teresa Liliana Wargasetia, Nurhalim Shahib, Djamhoer Martaadisoebrata, Diah Dhianawaty, Bethy Hernowo

**Affiliations:** 1 *Faculty of Medicine, Maranatha Christian University Jl. Prof. Drg. Suria Sumantri MPH No. 65, Bandung 40164, Indonesia.*; 2 *Faculty of Medicine, Padjadjaran University Jl. Eijkman No. 38 Bandung 40161 Indonesia.*

**Keywords:** *Gestational trophoblastic disease*, *Apoptosis*, *Autophagy*, *Bcl*-*2*, *Beclin*-*1*

## Abstract

**Background:**

The pathogenesis of Gestational Trophoblastic Disease (GTD) is not clearly known.

**Objective:**

In this study, immunoexpression of proteins Bcl-2 and Beclin-1 in trophoblastic lesions and normal trophoblastic tissue was conducted to study the mechanism of apoptotic and autophagic cell death that is expected to complete the study of GTD pathogenesis.

**Materials and Methods:**

Bcl-2 and Beclin-1 immunoexpression were studied on complete hydatidiform mole, partial hydatidiform mole, invasive mole, choriocarcinoma and normal placenta slides.

**Results:**

The average total scores of Bcl-2 immunoexpression had a decreasing value, starting from partial hydatidiform mole (3.09), complete hydatidiform mole (2.36), invasive mole (1.18) to choriocarcinoma (0) when compared to normal placenta (6). The results showed no significant difference in Beclin-1 immunoexpression total score between complete hydatidiform mole, partial hydatidiform mole and invasive mole, namely that the value of the average total score of Beclin-1 was low (2.27, 2.45 and 2.36), but on the contrary choriocarcinoma showed an increasing strong Beclin-1 expression with the average total score of 4.57.

**Conclusion:**

Bcl-2 expression decreases in line with the excessive proliferation of trophoblast cells in hydatidiform mole and leads to malignancy in invasive mole and choriocarcinoma. The decreased expression of Beclin-1 that leads to autophagy defects in complete hydatidiform mole, partial hydatidiform mole and invasive mole shows the role of autophagy as tumor suppressor, whereas strong Beclin-1 expression shows the survival role of autophagy in choriocarcinoma. The change of Bcl-2 activity as antiapoptosis and Beclin-1 as proautophagy plays a role in pathogenesis of GTD.

## Introduction

Benign hydatidiform moles and malignant gestational trophoblastic tumors are included in a group (spectrum) of diseases, namely gestational trophoblastic diseases (GTD). Hydatidiform mole consists of two types, namely complete hydatidiform mole and partial hydatidiform mole, whereas gestational trophoblastic tumor (GTT) is classified into invasive mole, choriocarcinoma, placental site trophoblastic tumor, and epithelioid trophoblastic tumor based on anatomic pathology examination (1, 2)*.*

 In developed countries, the incidence of hydatidiform mole and GTT is low. The diseases are more common in Asian and Latin-American countries. One of the problems in GTD is that most of the hydatidiform mole will undergo spontaneous regression after curettage, but 8-30% of patients suffer from GTT in the future and need chemotherapy. Until now the prognostic factor used has been the serial assay β-human chorionic gonadotropin (β-HCG) level of the serum measured during follow-up after evacuation. There are no available genetic or other molecular markers to predict as early as possible the aggressive behavior of hydatidiform mole (3, 4).

This research is aimed to study the pathogenesis of GTD by highlighting the mechanisms of programmed cell death. Nowadays, programmed cell death is a field with the most rapid progress in cancer research and has become the center of attention in cancer therapy (5).

Programmed cell death includes apoptosis (type I), autophagy (type II), and necrosis (type III) (6). Apoptosis is important to get rid of cancer cells so it is an important mechanism for tumor suppression (7, 8). Autophagy involved in tumor suppression and defects in autophagy contribute to oncogenesis (8-12).

B-cell lymphoma 2 (Bcl-2) is the first cellular protein identified to function as an oncogene protein by blocking apoptotic cell death (7). Bcl-2 gene was cloned for first time from the breakpoint t (4:18) chromosome translocation of follicular lymphoma of patients. Bcl-2 gene is located in human chromosome 18q21 (13). Various studies on Bcl-2 immunoexpression on the PTG show various results (14).

Beclin-1 gene (ortolog of mammals from Atg6 in yeast), also known as BECN1, is the key regulator of autophagy genes (11, 13, 15). Beclin-1 gene is found in human chromosome 17q21 (16, 17). In mice, heterozygous mutations of Beclin-1 gene increase cell proliferation and cancer development, but Beclin-1 is expressed in wild type (18, 19). Monoalelic deletion of Beclin-1 gene is found in 40-75% of sporadic breast r, ovarian, and prostate cancers (18). Several studies have shown a decrease of Beclin-1 protein immunoexpression in a number of cancers (10, 16, 20-26), but several other studies have detected an increase of Beclin-1 protein expression in cancer compared to normal tissues (11, 27, 28).

This study seeks to find out Bcl-2 and Beclin-1 immunoexpressions at GTD that are expected to contribute to the study of the pathogenesis of trophoblastic lesions that provide the basis for pathological and molecular diagnosis of various types of trophoblastic tumors and the search for prognosis genetic markers.

## Materials and methods

In this cross-sectional study Tissue samples evacuated from the archive with reconfirmed complete hydatidiform moles (n=11), partial hydatidiform moles (n=11), invasive moles (n=11) and choriocarcinomas (n=9) were studied. Placental specimens from normal pregnancies (n=6) were included. The slides were stained with immunohistochemical technique with mouse anti-Human oncoprotein Bcl-2a monoclonal antibody (Biocare Medical, AM 003) and rabbit anti-Human Beclin-1 monoclonal antibody (EPR1733Y Abcam, ab51031).

Bcl-2 and Beclin-1 immunoexpressions were analyzed semiquantitatively based on the score value of staining intensity, which was 0 if there were no stained cells, 1 for weak staining, 2 for moderate staining, and 3 for strong staining (10, 28-31). In addition to the intensity scores, immunoexpression was also analyzed by determined score distribution, that is a score of 0 if there were no stained cells, 1 for <30% of stained cells, 2 for 30-60% of stained cells, and 3 for > 60% of stained cells. The total score of immunoexpression was obtained from the value of the sum of intensity score and distribution score (25).

The study was conducted in the Anatomical Pathology Department, Faculty of Medicine, Padjadjaran University/Hasan Sadikin Hospital Bandung after receiving approval from the Health Research Ethics Committee of the Faculty of Medicine, University of Padjadjaran, Bandung, Indonesia.

**Statistical analysis**

Data were analyzed by means of the Kruskal-Wallis test, followed by Mann-Whitney test. The significance of the test results was determined when p≤0.05. SPSS 17.0 software (Statistical Package for the Social Sciences, version 17.0, SPSS Inc, Chicago, Illinois, USA) was used to analyze the data.

## Results

**Figure 1:**
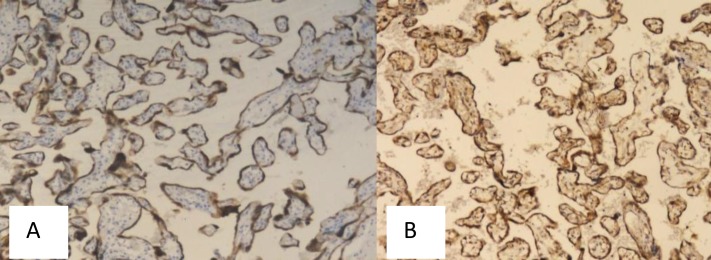
Bcl-2 (A) and Beclin-1 (B) Immunoexpression, Score 6 (Strongly Positive), Magnification 100x, in Normal Placenta

**Figure 2:**
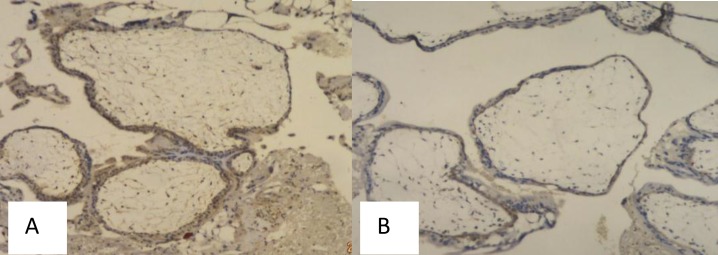
Bcl-2 Immunoexpression, Score 4 (Moderately Positive) (A) and Beclin-1 Immunoexpression, Score 2 (Weakly Positive) (B), Magnification 100x, in Partial Hydatidiform Mole

**Figure 3:**
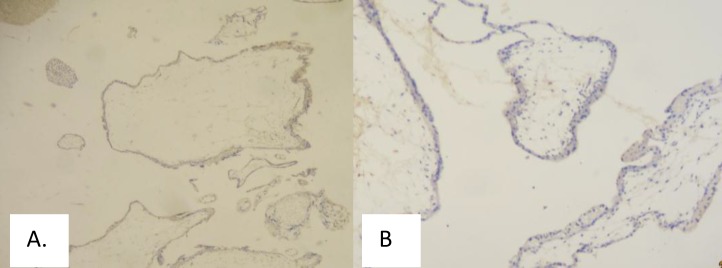
Bcl-2 Immunoexpression, Score 2 (Weakly Positive), Magnification 40x (A) and Beclin-1 Immunoexpression, Score 2 (Weakly Positive), Magnification 100x (B), in Complete Hydatidiform Mole

**Figure 4:**
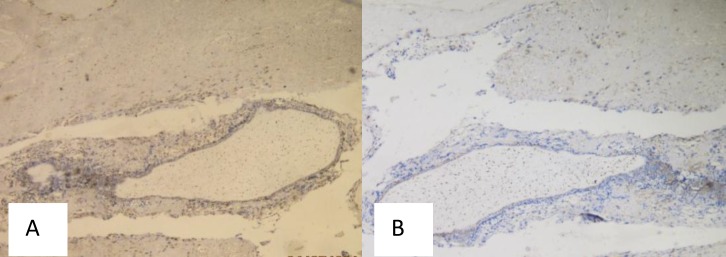
Bcl-2 (A) and Beclin-1 (B) Immunoexpression, Score 2 (Weakly Positive), Magnification 40x, in Invasive Mole

**Figure 5:**
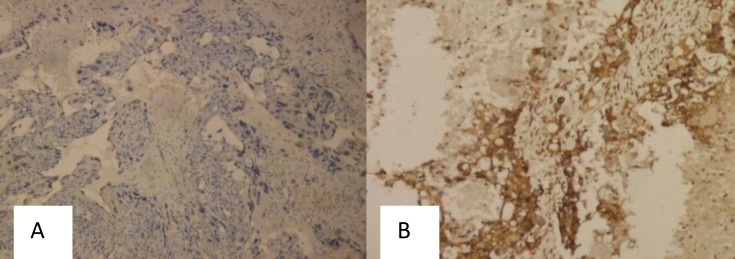
Bcl-2 Immunoexpression, Score 0 (Negative) (A) and Beclin-1 Immunoexpression, Score 6 (Strongly Positive) (B), Magnification 100x, in Choriocarcinoma

The results showed that Bcl-2 and Beclin-1 were expressed in the cytoplasm of trophoblast cells in GTD as well as in normal placenta (Figure 1-5).

**Figure 6:**
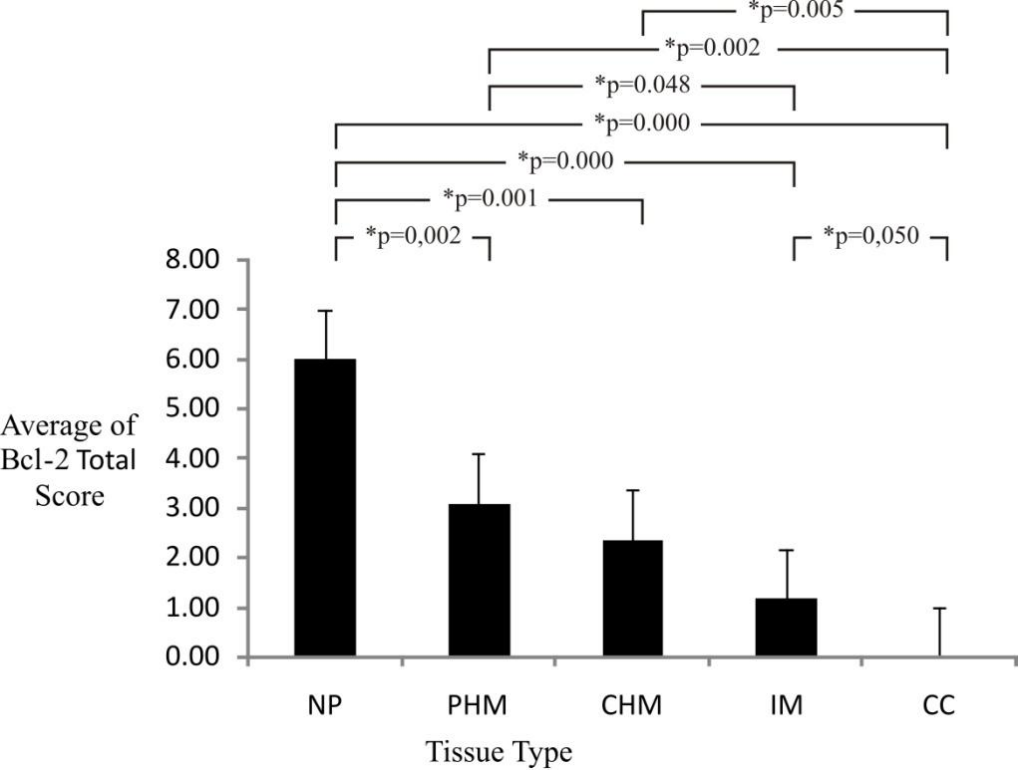
Calculation Result of Average and Mann-Whitney Test for Bcl-2 Immunoexpression Total Score in Gestational Trophoblastic Disease and Normal Placenta

The research results showed a decrease in the total score averages of Bcl-2 immunoexpression which was gradual from normal placenta that showed strong immunoexpression with maximal score (6), then partial hydatidiform mole (3.09), complete hydatidiform mole (2.36), invasive mole (1.18), until choriocarcinoma with negative immunoexpression (score 0) (Figure 6).

**Figure 7:**
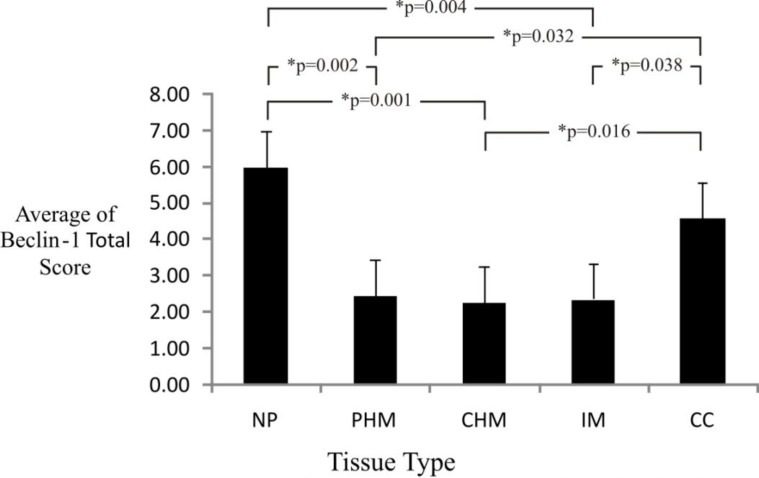
Calculation Result of Average and Mann-Whitney Test for Beclin-1 Immunoexpression Total Score in Gestational Trophoblastic Disease and Normal Placenta

The total score averages of Beclin-1 immunoexpression for partial hydatidiform mole, complete hydatidiform mole and invasive mole were 2.5, 2.27 and 2.36. The results of Mann-Whitney test showed that there was no significant difference of Beclin-1 immunoexpression between partial hydatidiform mole, complete hydatidiform mole and invasive mole (p>0.05). The three molar types had significantly different Beclin-1 immunoexpression from choriocarcinoma (p = 0.032, p = 0.016, p = 0.038, respectively). The total score average of Beclin-1 immunoexpression for choriocarcinoma was 4.57. The entire samples of normal placental showed a strongly positive expression of Beclin-1, so the maximal average of immunoexpression total score was obtained, namely was 6 (Figure 7). The results of Mann-Whitney test to Beclin-1 immunoexpression in choriocarcinoma and normal placenta showed no significant difference between the two of them (p>0.05). In line with that the three molar types had significantly different Beclin-1 immunoexpression from normal placenta (p = 0.001, p = 0.002, p = 0.004, respectively).

NP= Normal placenta, PHM= Partial Hydatidiform Mole, CHM= Complete

Hydatidiform Mole, IM= Invasive Mole, CC= Choriocarcinoma

NP= Normal Placenta, CHM= Complete Hydatidiform Mole, PHM= Partial

Hydatidiform Mole, IM= Invasive Mole, CC= Choriocarcinoma

## Discussion

Bcl-2 is a protein that is known to have a role to inhibit apoptosis. Based on the calculation of immunoexpression total score, the total score average of Bcl-2 immunoexpression for complete hydatidiform mole is 2.36, weaker than normal placenta which shows strong Bcl-2 expression (total score average is 6). This finding is in line with the publication of Mochizuki *et* *al.* in 1998, Qiao *et al*. (1998) and Hussein (2009) (31-33).

Choriocarcinoma is a malignant disease characterized by abnormal trophoblast hyperplasia and anaplasia, absence of chorionic villi, hemorrhage, and necrosis, with direct invasion into the myometrium and vascular invasion that results in the spread to various organs of the body (2) (34). So, necrosis is one of the characteristics in choriocarcinoma. According to the literature written by MacManus and Linnik, Bcl-2 inhibits apoptosis and necrosis (35). In this study, expression of Bcl-2 in choriocarcinoma was weak, so it can be presumed that there was an increase in apoptotic cell death and necrosis.

From the research result shown in Figure 6, it is known that the total score averages of Bcl-2 immunoexpression become lower and lower, starting from partial hydatidiform mole, complete hydatidiform mole, invasive mole, until choriocarcinoma when compared with normal placenta. Bcl-2 is antiapoptosis, so the decrease in the expression of Bcl-2 indicates an increase in apoptosis. These results are consistent with some studies that show an increase in villous trophoblast apoptosis in placental pathology, including miscarriage, preeclampsia, intrauterine growth restriction, partial and complete hydatidiform mole and choriocarcinoma. In molar pregnancy, increased apoptosis reflects the fate of trophoblast cells as a result of uncontrolled hyperplasia (36, 37). Higher levels of apoptosis are observed in the more invasive and proliferative disease (37)*.* Changes in placental function due to external factors such as hypoxia and reactive oxygen species can lead to significant increase in apoptosis through decreased expression of Bcl-2, increased expression of p53 and Bax, and activation of Caspase -3 and 9 (37, 38).

Negative or weak expression of Bcl-2 in trophoblast cells may also be related to a microRNA that inhibits Bcl-2. So far studies have revealed that there are 30 kinds of microRNA associated with apoptosis, some microRNA acting as antiapoptosis and some other acting as proapoptosis (39). Cimmino *et* *al*. found that miR-15a and miR-16-1 suppress the expression of Bcl-2 resulting in the induction of apoptosis in leukemia cell lines (40).

There are researches which show that Bcl-2 protein is involved in cellular process besides apoptosis which influences the progression of tumor. Bcl-2 also has anti proliferation effect which inhibits cell cycle progression form dormant to S phase (41). The anti proliferation effect from Bcl-2 inhibits tumor progression on animals (41, 42). The loss of Bcl-2 expression is related to the increase of oral carcinoma proliferation activity (43). The same case can happen to GTD. The decrease of Bcl-2 expression is related to the progression of this disease because trophoblasts become responsive to mitotic stimuli (30).

Bcl-2 expression decreases in line with the excessive proliferation of trophoblast cells in hydatidiform mole, leading to malignancy in invasive mole and choriocarcinoma. Therefore, it can be said that the decrease in Bcl-2 indicates a poor prognosis. Decreased expression of Bcl-2 can also be used for early diagnosis of abnormalities of trophoblast cells with the proliferation tendency to rise as the expression of Bcl-2 decreases.

In this research the presence of Beclin-1 protein in GTD and normal placenta was also studied. Beclin-1 is a gene in mammals that was first identified to have a role in controlling autophagy (16). Autophagy is a protective process in the cell that is essential for the degradation and mitochondrial quality control, degradation and quality control of protein and cellular energy homeostasis (12). Therefore, Beclin-1 is required for autophagy process that plays a role in maintaining homeostasis within cells. The result of this study which show a strong expression of Beclin-1 in all samples of normal placenta strengthens the theory about the important role of Beclin-1 in the metabolism process of normal cells.

It is known that there were no Beclin-1 immunoexpression significant differences between complete hydatidiform mole, partial hydatidiform mole and invasive mole. In the three types of mole, the total score averages of Beclin-1 immunoexpression were low (Figure 7). These results are consistent with numerous studies that decreased expression of Beclin-1 protein is associated with abnormalities in breast cancer cells, cervical squamous cell carcinoma, brain tumor, ovarium cancer, hepatocellular carcinoma, lung cancer, and esophageal squamous cell carcinoma (10, 16, 20-26).

Liang’s research demonstrated that the autophagy gene expression inhibits the potential tumor formation. This indicates that autophagy is the basic mechanism to prevent the growth of tumor cells. A decrease of autophagy will increase oxidative stress and the accumulation of mutations in precancerous cells. Numerous studies confirm that Beclin-1 is a tumor suppressor gene that shows decreased expression in various tumor tissues (10, 16, 20-26). So it can be assumed that in the three types of mole, a decrease in Beclin-1 causes abnormalities in the autophagy process so that trophoblast cells death is inhibited, while the proliferation of trophoblast cells continue to occur, causing hyperplasia of cytotrophoblast and syncytiotrophoblast cells.

There is controversy concerning the role of autophagy in cancer. A number of studies show that autophagy suppresses tumor development, whereas a number of other studies convey that autophagy supports tumor development and protects the tumor cells from cell death stimuli (9, 11, 25, 44, 45). The nature of this paradox is known in the expression that “autophagy is a double-edged sword in oncology” (46).

In this study, the paradox role of autophagy in cancer was found. The total score averages of Beclin-1 immunoexpression were low in complete hydatidiform mole, partial hydatidiform mole, and invasive mole, and there were no significant differences of Beclin-1 immunoexpression total score between the three types of mole (p>0.05). On the other hand, the strong Beclin-1 expression was dominant in choriocarcinoma with total score average of 4.57 ~ 5, so that it had a significantly diffferent Beclin-1 immuno-expression total score (p<0.05) from the three types of mole (Figure 7). This condition supports the hypothesis made by experts that autophagy mediates damaged cells treatment that suppresses tumor initiation in precancerous cells and promotes survival in established cancer. This paradox occurs because autophagy is the mechanism responsible for the degradation and recycling of various intracellular macromolecules as well as the destruction of damaged cell organelles by lysosomes. On the one hand, autophagy overcomes DNA damage and genomic instability and causes cell death so that it has the nature as a tumor suppressor. The presence of mutations in the autophagy gene causes low level autophagy in tumor cells. Tumor cells are exposed in metabolic increase and other stresses which make these cells depend on autophagy because in the process of autophagy amino acids and free metabolites are released to be used for survival (47). Decreased expression of Beclin-1 leads to defects of autophagy in early tumorigenesis in certain cancers, while increased expression of Beclin-1 is a characteristic of an increase in autophagy survival in established tumors (20).

Microscopically, choriocarcinoma show cells with the appearance like monuclear cytotrophoblast and in other areas are seen cells like syncytiotrophoblast cells with many nuclei and highly pleomorphic. Transformation of trophoblast cells into malignant trophoblastic hyperplasia is characterized by abnormal hyperplasia trophoblas, anaplasia, invasion into the myometrium, and vascular invasion that leads to metastasis (34). In choriocarcinoma cells that are established tumors, autophagy is maintained as an attempt to survive as indicated by the strong expression of Beclin-1. From the literature search, it is found that increased protein Beclin-1 expression occurs in intrahepatic cholangiocarcinoma, colorectal cancer, and gastric cancer (11, 27, 28).

In contrast to the weak expression of Beclin-1 with low total score averages of immunoexpression in moles, there is peculiarity in choriocarcinoma in the form of strong expression of Beclin-1 with a high total score average. This indicates the Beclin-1 expression potential as a tumor marker for diagnosis and determination of prognosis towards choriocarcinoma.

## Conclusion

1. Bcl-2 expression decreases in line with the excessive proliferation of trophoblast cells in hydatidiform mole and leads to malignancy in invasive mole and choriocarcinoma.

2. The decreased expression of Beclin-1 that leads to autophagy defects in complete hydatidiform mole, partial hydatidiform mole and invasive mole shows the role of autophagy as tumor suppressor, whereas strong Beclin-1 expression shows the survival role of autophagy in choriocarcinoma.

3. The change of Bcl-2 activity as antiapoptosis and Beclin-1 as proautophagy plays a role in pathogenesis of gestational trophoblastic diseases.

## Note

**This article extracted from Ph.D. thesis. (Teresa Liliana Wargasetia)**
